# Dried Figs Quality Improvement and Process Energy Savings by Combinatory Application of Osmotic Pretreatment and Conventional Air Drying

**DOI:** 10.3390/foods10081846

**Published:** 2021-08-10

**Authors:** Varvara Andreou, Ioanna Thanou, Marianna Giannoglou, Maria C. Giannakourou, George Katsaros

**Affiliations:** 1 Institute of Technology of Agricultural Products, Hellenic Agricultural Organization-DEMETER, Lykovrissi, 14123 Attica, Greece; vandreou@chemeng.ntua.gr (V.A.); giannoglou@chemeng.ntua.gr (M.G.); 2 Department of Food Science and Technology, University of West Attica, Egaleo, 12243 Attica, Greece; ioannavthanou@gmail.com (I.T.); mgian@uniwa.gr (M.C.G.); 3 Institute of Technology of Agricultural Products, ELGO-DEMETER, Sof. Venizelou 1, Str., 14123 Lykovrissi, Greece

**Keywords:** figs, osmotic dehydration, air-drying, energy consumption, quality, shelf-life

## Abstract

This study concerns the implementation of osmotic dehydration (OD) as a pre-treatment of air-drying in fig halves, aiming at drying acceleration, energy savings and product quality improvement. The effect of solid/liquid mass ratio, process temperature (25–45 °C) and duration (up to 300 min) on water activity (a_w_) and transport phenomena during OD, was modelled. The effective diffusion coefficients, drying time and energy consumption, were also calculated during air-drying at 50–70 °C. At optimum OD conditions (90 min, 45 °C), the highest water loss and solid gain ratio were achieved, while the a_w_ (equal to an initial value 0.986) was decreased to 0.929. Air-drying time of OD- and control samples was estimated at 12 and 21 h, at 60 °C, respectively, decreasing the required energy by up to 31.1%. Quality of dried figs was systematically monitored during storage. OD-assisted air-drying led to a product of improved quality and extended shelf-life.

## 1. Introduction

Figs (*Ficus carica* L.) are fruits of superior nutritional quality, widely consumed mainly in Greece, Turkey, Italy, and Algeria [1]. Rich in several vitamins and also an important source of carbohydrates [2], figs are mainly consumed fresh. However, due to their high moisture content which promotes microbial growth, they are very perishable, making it necessary to prolong their short post-harvest life [2].

Air-drying is a food preservation method that is based on moisture removal, resulting in a substantial water activity (a_w_) decrease, limited microbial activity and minimum enzymatic and chemical reactions rates [3]. However, in the case of high moisture food products, the decrease in their a_w_ requires the application of high temperatures for long times, resulting in quality degradation (shrinkage, hardening, browning, degradation of sensory characteristics) and decreasing consumer acceptability. At the same time, the increased energy costs make air-drying a cost-intensive food preservation process [4]. The disadvantages of the conventional air-drying and the requirement for more efficient fruit processing methods have led to the investigation of alternative dehydration techniques [5,6,7]. The osmotic dehydration (OD) process comprises the partial water removal of a food through its immersion in a hypertonic solution [8]. The diffusion phenomenon takes place with two countercurrent flows: a water flow from the food to the outer solution and a simultaneous flow of solute from the solution to the food [9], enriching its composition and thus leading to significantly enhanced nutritional value. These mechanisms lead to water loss and solid gain within the food. OD process usually takes place at mild temperatures (<50 °C); thus, low energy consumption and processing costs are required [10].

Current research is focused on the use of air-drying process assisted by OD, indicating enhanced water transport phenomena, improved food quality and decreased energy consumption, mainly due to the use of milder air-drying conditions [11]. Several researchers have studied the use of OD as a pretreatment to air-drying for fruits and vegetables such as tomato and cucumber [12], goji berry [13], apple [14], banana [15], pumpkin [16] and melon [17]. The beneficial use of OD has also been demonstrated for dairy [18] and meat products [19]. OD could also be efficiently applied in the case of produce already available in the market, e.g. dried figs that have quality issues such as increased hardness and shrinkage, due to their long stay in high drying temperatures.

The effect of various osmotic solutions on the mass transfer phenomena of OD treatment and on the quality of figs, with OD being a stand-alone dehydration technique, is being reported [1,9,20,21,22]. There are limited works that have studied the combined effect of OD with air-drying [23,24] on the quality of final dried figs. Our work differs from other studies cited in the literature, since it provides not only experimental data but also mathematical description of the effect of both osmotic dehydration and air-drying techniques (no work presents relevant information on a tissue such as fig) on the quality of fig halves. In addition, the effect of process conditions on the quality of the final dried products was studied, evaluating quality and nutritional indices necessary for their characterization. The potential reuse of OD solution is also described. A shelf-life study to validate the effectiveness of combined OD and air-drying techniques, in comparison to the conventionally air-dried fig tissue, was conducted. The energy savings were estimated and a cost analysis (based on the process optimization performed for OD and air-drying techniques) is also provided, offering a holistic approach of how these two combined techniques may have a significant impact on the drying of figs.

## 2. Materials and Methods

### 2.1. Raw Material

The study was carried out with figs of “*Markopoulo Royal Black*” variety, cultivated and harvested in the region of Markopoulo (Attica, Greece). After harvesting, the figs were grouped into three ripening stages (RS) based on their skin color and dimensions (RS I: green skin; <6 cm diameter, RS II: green-purple skin; <8 cm diameter, RS III: dark purple skin; <10 cm diameter) [25]. All experiments were performed using figs of the first ripening stage, while their initial moisture content was approximately 81.2 ± 2.2%, ensuring that have roughly the same sugar content. The a_w_ value of the fresh figs was measured as 0.986 ± 0.003.

### 2.2. Osmotic Dehydration (OD) Pretreatment

#### 2.2.1. Experimental Design

Fig halves were osmotically dehydrated in a multi-component solution containing (*w*/*w*) 80% glycerol, 1% wine vinegar, 0.5% ascorbic acid, 1.0% sodium chloride, a formula selected based on preliminary experiments on the effect of different formulas of OD solution on the a_w_ and sensory characteristics of the product. The advantage of a multi-component solution is that the final osmo-dehydrated sample would have a combination of characteristics obtained from each solute. The selection of the ingredients was based on the high rate of mass transfer phenomena during osmosis and on the high-quality with low water activity of the final osmo-dehydrated samples. There are many osmotic agents that are commonly used in the literature, such as sucrose, fructose or sugar. In this study, the main osmotic agent (80%) of OD solution was food-grade glycerol. Its low molecular weight resulted in a significant reduction of water activity of the OD solution, enhancing the mass transfer phenomena during osmosis. Glycerol is also convenient, non-toxic and is considered as sugar substitute sweetener that does not react with the product. Moreover, wine vinegar (1%) was selected as ingredient for its antimicrobial properties, also causing reduction in pH-value of OD solution, necessary when the final product has increased sweet mouth-feeling. Sodium chloride (1%) was added, also aiming to sensory improvement of final product in order to balance the sweet taste of glycerol. Ascorbic acid (0.5%) addition aimed to increased antioxidant activity and enzymatic browning prevention during OD.

OD was carried out at three different temperatures (25, 35 and 45 °C), two different mass ratios of the fruit to OD solution (1/4 and 1/6 (*w*/*w*)), under shaking (160 rpm) and for processing times up to 300 min. During OD, figs were removed at predefined times for further analysis. All analyses were carried out in duplicate.

#### 2.2.2. Evaluation of Osmotic Pretreatment Progress

Τhe effect of the processing conditions (temperature, time and solid to liquid ratio) on the OD progress and the evolution of the mass transfer phenomena were evaluated, through the determination of selected parameters. Water loss (WL) (g H_2_O/g initial dry matter (i.d.m.)) and solid gain (SG) (g solids/g i.d.m.) of the OD-treated fig halves, were calculated using Equations (1) and (2), respectively:

(1) $$\mathrm{WL}=\frac{\left(M_{0}-m_{0}\right)-\left(M-m\right)}{m_{0}}$$

(2) $$\mathrm{SG}=\frac{\left(m-m_{0}\right)}{m_{0}}$$

where *M*_0_ is the initial mass of the sample (g), *m*_0_ is the initial dry mass (g), *M* is the final mass of the sample at time *t* of OD (g), and *m* is the dry mass of solids at time *t*of OD (g).

Moisture content was determined by drying the samples at 110 °C (Memmert, B50 type, Memmert GmbH + Co. KG) for 24 h. During OD, the a_w_ of the fig halves was monitored at 25 °C using a water activity measurement device (Aqua LAB 4TEV, Decagon, 2365 NE Hopkins Ct. Pullman, WA 99163, USA). OD progress was also evaluated by means of color (CIELab values; CR-300 Minolta Chromameter, Minolta Co., Chiyoda-ku, Tokyo 100-7015, Japan), texture analysis (TA.HDplus, Stable Micro Systems Ltd., Vienna Court, Lammas Rd, Godalming GU7 1YL, UK) and sensory evaluation (color, texture, flavor) of the fig halves.

The selection of the optimal OD processing conditions for the fig halves was based on the maximum mass transport phenomena (WL and SG) rates and the highest a_w_ reduction combined with the optimal sample quality.

#### 2.2.3. Reuse of Osmotic Solution

Aiming to present OD technique as more environmentally friendly in terms of waste reduction and reduced running costs, reconstitution of the OD solution was carried out for four consequent trials. OD took place at optimal conditions (T: 45 °C, t: 90 min, ratio: ¼) using the reconstituted solution. The values of WL, SG and a_w_ of the fig halves of each OD treatment at the optimal processing time (90 min), were determined for the four times reconstituted solutions and compared to the results obtained from the initial trial of OD.

The obtained OD solution at the end of the fourth trial was microbiologically analyzed. Total viable count (TVC) (Plate Count Agar-Biokar Diagnostics, Beauvais, France) and yeasts and molds (Rose-Bengal Chloramphenicol Agar-Biokar Diagnostics, Beauvais, France) were determined using the surface plating technique (ISO4833-2:2013).

### 2.3. Air-Drying Process

Air-drying was carried out using a conventional air-dryer (SousVideTools Hendi 229002 6-Tray Shelf Fruit Dryer). OD-treated (at the optimal OD conditions: T: 45 °C, t: 90 min, ratio 1/4) and untreated (control) figs were air-dried at three different temperatures (50, 60 and 70 °C) for durations up to 20 h. During air-drying, the samples were a_w_ analyzed and weighed every 30 min, in order to estimate the water removal from the figs, until a_w_ reached a value of 0.55. Total air-drying time was defined as the necessary time (t_DR_) to achieve an a_w_-value equal to 0.55–0.60 for the studied air-drying temperatures for both OD-treated and untreated samples.

### 2.4. Monitoring of Quality Characteristics

The OD-pretreated and air-dried figs were evaluated in terms of quality (chroma and texture), nutritional parameters, bioactive compounds, and sensory characteristics.

#### 2.4.1. Chroma and Texture Analysis

The firmness of the samples was measured using a ΤA.HD Plus texture analyzer (Stable Micro Systems Ltd., Godalming, UK). The samples were cut using a knife probe. The speed and depth were set equal to 0.5 mm/s and 3 mm, respectively. The maximum peak force (F_max_, N) was the mechanical parameter measured, and it was expressed as firmness.

The color was measured using a colorimeter Minolta CR-300 (Minolta Company, Chuo-Ku, Osaka, Japan). The measurements were expressed in CIELab color scale (Commission International de l’ Eclairage). The total color change ΔE was calculated according to Equation (3):

(3) $$\Delta E=\sqrt{{\left(L^{*}{}_{t}-L^{*}{}_{0}\right)}^{2}+{\left(a^{*}{}_{t}-a^{*}{}_{0}\right)}^{2}+{\left(b^{*}{}_{t}-b^{*}{}_{0}\right)}^{2}}.\;$$

where Δ*E* is the color change, L*, a* and b* are the luminosity, redness and yellowness of the samples, respectively. Subscripts “*t*” and “0” refer to time *t*and zero time, respectively. All measurements were replicated at least five times.

#### 2.4.2. Analysis of Nutritional Compounds

Total protein content of all samples was determined according to Kjeldahl method (IDF, 2008) using a Kjeldahl rapid distillation unit (Protein Nitrogen Distiller DNP-1500-MP, RAYPA, 08227 Terrassa, Barcelona, Spain). Total fiber content of both samples was measured by applying the Weende Method (AOAC 984.04) with some modifications [26] using a manual crude fiber analyzer (FibreBag-Fibretherm, Gerhardt analytical systems, 53639 Königswinter, Germany).

Individual sugars (fructose, glucose, and glycerol) and organic acids (citric, mallic, tartaric and lactic) were determined by using a high-performance liquid chromatography method as described by Sturm, Koron, and Stampar [27].

Individual sugars and organic acids were quantified according to calibration curves performed with standard solutions and their concentrations were expressed as g/100 g dry weight (d.w.).

L-ascorbic acid of figs was determined by a titrimetric method using 2,6-dichlorophenolindophenol (DCIP) as standard solution (AOAC 1984, 43,064). Results were expressed as mg ascorbic acid per 100 g dry weight (mg AA/100 g d.w). All measurements were replicated twice.

#### 2.4.3. Determination of Bioactive Compounds

The concentration of total polyphenols (mg caffeic acid equivalents (CAE)/100 g d.w.), total flavonoids (mg catechin/100 g d.w.) and the antioxidant capacity (mg Trolox/100 g d.w.) of the dried figs, were evaluated. The bioactive compounds extraction from the fig halves was carried out with a liquid-to-solid ratio (10:1) at ambient temperature according to Andreou, Psarianos, Dimopoulos, Tsimogiannis, and Taoukis [28]. The total phenolic compounds were assessed using the Folin-Ciocalteu phenol reagent method [29]. The concentration of the extracts in total flavonoids was assessed according to Marinova, Ribarova, and Atanassova [30]. Antioxidant capacity of the extracts was determined according to the method described by Andreou et al. [28]. All above measurements were performed twice and their standard deviations were also calculated.

#### 2.4.4. Sensory Evaluation

Sensory evaluation of fig halves either during OD treatment or air-drying was carried out by 8 trained panelists. The samples were scored for their intensity in color, appearance, texture, taste, flavor and overall impression, in a scale of 0–9. The mean values were calculated for every characteristic.

### 2.5. Accelerated Shelf-Life Determination

OD-pretreated (at optimal selected OD conditions) and untreated fig halves were dried at 60 °C until a_w_ reached a value of 0.55. The figs were packed in glass containers and stored in high-precision incubators (Friocell 222-ECO line, MMM Group, Medcenter Einrichtungen GmbH, Planegg, Germany) at temperatures 25, 35, and 45 °C under darkness for more than 2 months. Based on quality their deterioration (color, firmness and sensory evaluation), the shelf-life of the figs for storage temperatures lower than 35 °C was determined by extrapolating the obtained results.

### 2.6. Data Analysis

#### 2.6.1. Mathematical Modelling of Osmotic Dehydration

The effect of OD conditions on water/solid transfer phenomena was mathematically modelled. Based on Fick’s 2nd law for diffusion and considering an infinite slab being dehydrated from both sides, the effective coefficients of water (*D_ew_*) and solids diffusivity (*D_es_*) were calculated by fitting the experimental measurements to Equations (4) and (5), supposing appropriate assumptions and boundary conditions [31]:

(4) $$M_{OD}=\frac{\left(WL_{t}-WL_{\infty }\right)}{\left(WL_{0}-WL_{\infty }\right)}=\frac{8}{\pi^{2}}\overset{\infty }{\underset{n=0}{\sum }}\frac{1}{{\left(2n+1\right)}^{2}}exp\left[-{\left(n+\frac{1}{2}\right)}^{2}\pi^{2}D_{ew}\frac{t}{l^{2}}\right]$$

(5) $$S_{OD}=\frac{\left(SG-SG_{\infty }\right)}{\left(SG_{0}-SG_{\infty }\right)}=\frac{8}{\pi^{2}}\overset{\infty }{\underset{n=0}{\sum }}\frac{1}{{\left(2n+1\right)}^{2}}exp\left[-{\left(n+\frac{1}{2}\right)}^{2}\pi^{2}D_{es}\frac{t}{l^{2}}\right]$$

where *M_OD_* and *S_OD_* are the dimensionless water loss and solid gain ratios, respectively, *D_ew_* and *D_es_* are the effective diffusivity coefficients for water loss and solid gain, respectively and *l* the slab half thickness.

#### 2.6.2. Mathematical Modelling on the Air-Drying Processing of the Figs

To assess the progress of figs air-drying, drying curves were constructed using the dimensionless moisture ratio (*ω*) (Equation (6)):

(6) $$\omega =\frac{{Μ}_{t}-M_{0}}{{Μ}_{\infty }-M_{0}}.\;$$

where *ω* is the dimensionless moisture ratio, *M_t_* is the dry basis moisture content at drying time *t*, *M_∞_* is the moisture content that corresponds to the respective moisture for selected a_w_ value of dried figs (~0.55), and *M*_0_ is the initial moisture content (on a dry basis).

Drying curves were described by Fick’s second law of diffusion for an infinite slab [32], (Equation (7)):

(7) $$\omega =\frac{8}{\pi^{2}}\overset{5}{\underset{n=0}{\sum }}\frac{1}{{\left(2n+1\right)}^{2}}exp\left[-{\left(n+\frac{1}{2}\right)}^{2}\pi^{2}D_{eff}\frac{t}{l^{2}}\right]\;$$

where *ω* is the dimensionless moisture ratio, *D_eff_* is the effective moisture diffusion coefficient (m^2^/s), and *l* is the slab half thickness (m).

The dependence of the air-drying temperature on the effective moisture diffusion coefficient of both samples was expressed through the activation energy (*E_a_*), calculated using the Arrhenius equation (Equation (8)):

(8) $$lnD_{eff}=lnD_{eff\left(Tref\right)}+\frac{E_{a}}{R}\cdot \left(\frac{1}{T}-\frac{1}{T_{ref}}\right)\;$$

where *D_eff_* and *D_eff_*(*T_ref_*) are the effective moisture diffusion coefficients (m^2^/s) at *T* and *T_ref_* (55 °C) drying temperatures (K), respectively, *E_a_* is the activation energy (J/mol), and *R* is the universal gas constant (=8.314 J/mol K).

#### 2.6.3. Evaluation of Energy Consumption during Air-Drying

Air-drying energy consumption (*W_DR_*) was evaluated for both untreated and OD pretreated dried samples, in order to assess the potential energy savings obtained from the use of OD as a pretreatment. The *W_DR_* was calculated according to Equation (9) [33]:

(9) $$W_{DR}=\frac{A\cdot v\cdot \rho_{air}\cdot t_{AD}\cdot \Delta T\cdot C_{p}}{m_{0}}\;$$

where *W_DR_* is the drying energy consumption (MJ kg^−1^), *A* is the area of the container in which sample was placed for drying (m^2^), *v* is the air velocity (m s^−1^), *ρ_air_* is the air density at each drying temperature and atmospheric pressure (1 atm) (kg m^−3^), *t_AD_* is the total drying time (min), Δ*T* is the temperature difference (K), *C_p_* is the air specific heat at each drying temperature at atmospheric pressure (1 atm) (kJ kg^−1^ K^−1^), and m_0_ is the initial mass of figs (kg).

#### 2.6.4. Mathematical Modelling on the Shelf-Life Determination of the Figs

Total color change Δ*Ε* value (Equation (3)) during storage at all studied temperatures was modelled through apparent first-order kinetics, as described by equation (Equation (10)) and the shelf-life determination of the figs was calculated based on Equation (11):

(10) $$\ln\left(1-\frac{\Delta E}{\Delta E_{max}}\right)=k\cdot t$$

(11) $$t_{SL}=\frac{\ln\left(1-\frac{\Delta E_{limit}}{\Delta E_{max}}\right)}{k}$$

where Δ*Ε* is the color change at storage time *t*, Δ*Ε_limit_* (=20) is the color change, that was set as the average value for minimum acceptability, Δ*Ε_max_* (=25) the maximum Δ*Ε* value that was achieved after 6 months of storage, *k* the rate constant of the color change during storage (d^−1^) and, *t* the storage time (d).

### 2.7. Statistical Analysis

Each series of experiments was repeated twice, with all measurements being performed in -at least- duplicates the deviation among the data was expressed through standard deviation (mean value ± stdev). In the figures, the standard deviation among multiple treatments and measurements was represented through the use of error bars. The standard error of kinetic parameters, such as *D_eff_* (effective diffusion coefficient-Equation (7)) *E_a_* (activation energy-Equation (8)), *k* (rate constant of the color change during storage-Equation (10)), *t_SL_* (shelf-life determination-Equation (11)) and the goodness of fitting (R^2^) for each respective equation were determined by non-linear regression analysis. Duncan’s multiple range test was performed, to distinguish means with significant differences (*p* < 0.05), using Statistica 7 (Stat Soft, Tulsa, OK, USA).

## 3. Results

### 3.1. Osmotic Dehydration of Figs

**Figure 1:**
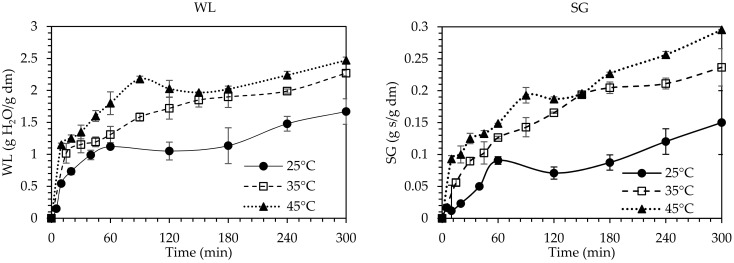
Water loss (WL) and Solid gain (SG) of figs during OD processing at temperatures 25 °C (⚫), 35 °C (☐) and 45 °C (▲) with 80% glycerol concentration and 1/4 ratio of sample per OD solution (*w/w*). Error bars represent standard deviation from multiple replications of treatments.

The effect of OD parameters (temperature and time) on the progress of the OD processing was investigated for 1/4 and 1/6 ratios of sample per OD solution (*w_s_*/*w_OD_*). The data obtained for the WL (Equation (1)) and SG (Equation (2)) of the figs during OD at 25, 35 and 45 °C were plotted (Figure 1).

WL and SG values increased significantly (*p* < 0.05) up to the first 100 min of OD for all studied samples, while for prolonged immersion times the process tended to equilibrium. Similar trends in the change of WL and SG have been reported in studies of OD on cherries [34], apple slices [35], and figs [9], where high mass transfer phenomena were observed till the first ~120 min, while for longer durations the system seemed to equilibrate. The highest values for WL and SG, observed at the end of the processing (300 min), were estimated as 1.67, 2.24 and 2.75 g H_2_O/g i.d.m. and 0.15, 0.23 and 0.29 g solids/g i.d.m. at 25, 35 and 45 °C, respectively. Higher OD temperature led to increased WL and SG values during OD. It was obvious that the mass transfer phenomena were more pronounced at 35 and 45 °C. A duration of 300 min of OD processing was necessary at 25 °C to reach WL and SG values equal to 1.67 g H_2_O/g i.d.m. and 0.15 g solids/g i.d.m., respectively, whereas at 45 °C, only 45 min was sufficient to achieve equal values of WL and SG. Dermesonlouoglou et al. [12,13] and Mandala et al. [14], reported that when increasing the OD temperature and time, the mass transfer phenomena are enhanced for many plant tissues, such as tomato (WL: 0.98–12.10 g H_2_O/g i.d.m.; SG: 0.05–4.52 g s/g i i.d.m.), cucumber (WL: 9.02–24.32 g H_2_O/g i.d.m.; SG: 0.08–4.95 g s/g i.d.m.), goji berries (WL: 1.12–2.48 g H_2_O/g i.d.m.; SG: 0.41–1.96 g s/g i.d.m.), and apple (WL: 0.55–1.65 g H_2_O/g i.d.m.; SG: 0.09–0.62 g s/g i.d.m.).

The experimental data obtained were fitted to Equations (4) and (5) and the respective effective diffusion coefficients (namely *D_ew_*, *D_es_*) were estimated (Table 1).

The obtained values of *D_ew_* and *D_es_* were in the ranges of 0.94–2.11 × 10^−10^ and 0.43–1.53 × 10^−10^ m^2^/s, respectively. No significant differences in *D_ew_* and *D_es_* values were observed between 1/4 and 1/6 *w_s_*/*w_OD_* (*p* > 0.05; Table 1). Moreover, the estimated *D_ew_* and *D_es_* values at 45 °C were two and three times higher, respectively, compared to their counterparts at 25 °C.

**Figure 2:**
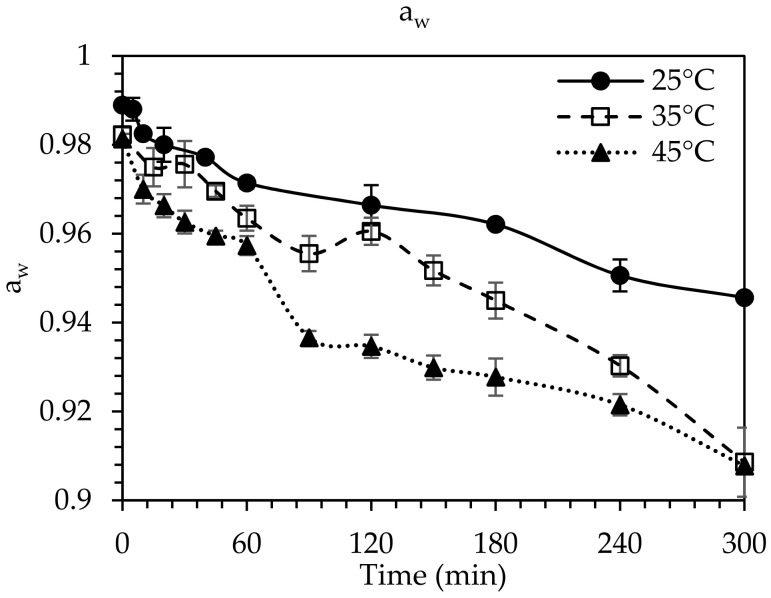
Water activity (a_w_) of the OD treated figs during OD processing at 25 °C (⚫), 35 °C (☐) and 45 °C (▲) with 80% glycerol concentration for 1/4 ratio sample per OD solution (*w_s_*/*w_OD_*). Error bars represent standard deviation from multiple replications of treatments.

During OD, a_w_ significantly decreased as time and temperature increased (*p* < 0.05) (Figure 2), which is mainly attributed to both water removal and solute uptake from the fig. This observation could be related to the initial low a_w_ value of the osmotic solution (0.5215). Processing at 25 °C led to a decrease in a_w_ of the figs from an initial value of 0.986 to ~0.946, while at higher temperatures (35 and 45 °C), a_w_ reached the value of ~0.907, at the end of OD (300 min) (Figure 2).

Regarding quality changes, a significant decrease in the redness of the fruit was observed, with a*-values ranging at the end of the OD from 10.41 ± 2.83 to 16.16 ± 2.84 depending on the intensity of the OD conditions, compared to control (23.32 ± 2.83) (*p* < 0.05). During OD, a slight decrease in the lightness of OD fig halves was also observed, with L*-values ranging from 31.51 to 38.76  ±  3.46 at the end of OD, while the corresponding L*-value of the untreated fig was 43.11  ±  3.46 (up to ~27% decrease). The color of the solution changed during OD, due to the leaching of pigments from the interior of the fig. Pereira, Ferrari, Mastrantonio, Rodrigues, and Hubinger [36] observed a decrease in the L*-values of tropical fruits during OD processing, attributing the phenomenon to the sugar gain. In contrast to these results, other researchers have reported that OD treatment increased the luminosity of fruits [13,37].

OD caused reduction in the firmness of the figs that was scored positively during sensory evaluation. At the end of OD, the firmness decreased from a value of 6.83 ± 1.32 N (untreated fruit) to 2.98 ± 1.84 N (~up to 56% decrease), independently of the intensity of the processing conditions (*p* > 0.05). This phenomenon could be attributed to cellular changes (plasmolysis) that took place, including loss of cell turgor and filling of air spaces with OD solution, resulting in disruption of the cell membranes [38]. Similar observations were reported by Najafi, Yusof, Rahman, Ganjloo, and Ling [39], Lewicki and Lukaszuk [38] and Castelló, Fito, and Chiralt [40] working on red pitaya, orange and strawberries, respectively. In contrary, there are studies that report increase of firmness of plant tissues after OD mainly attributed to the high solid gain caused by sugar impregnation [37,41]. Regarding fig shrinkage, no significant differences (*p* > 0.05) between OD pretreated and untreated samples were observed.

According to the sensory evaluation, OD treated samples received high scores on all organoleptic parameters, assessed as positive and desirable, thus confirming that the high glycerol concentration did not cause any negative effect on flavor and texture of the samples.

The selection of the optimal OD process conditions was based on a combination of a high a_w_ reduction, superior quality and as minimum processing duration as possible. A ratio of sample per OD solution 1/4 (*w_s_*/*w_OD_*), temperature 45 °C and processing time 90 min were selected as the optimal OD processing conditions. Under these conditions, OD samples exhibited adequate mass transfer of WL: 2.17 g w/g i.d.m., SG: 0.19 g s/g i.d.m. and an a_w_ decrease from ~0.9870 to ~0.9292.

#### Assessment of Reconstitution of OD Solution on the Effectiveness of OD Processing

The main bottleneck for the OD technique industrial scale-up, is the disposal of large quantities of osmotic solution, with potential environmental issues and high costs for the industry. The reconstitution of the diluted OD solution through the appropriate addition of solutes, could provide a partial solution to the problem.

**Figure 3:**
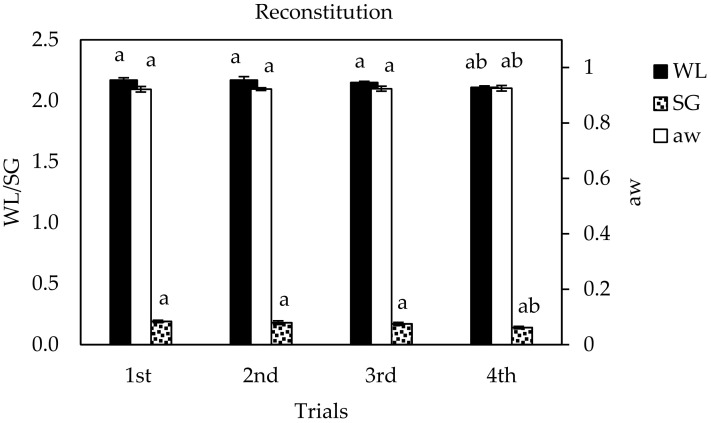
Comparison of a 3-times reconstituted OD solution (4 consecutively trials of OD treatments) on WL, SG and a_w_ of fig halves processed at optimal OD conditions (45 °C, 90 min, 1/4 ratio of sample per OD solution (*w_s_*/*w_OD_*)). Error bars represent standard deviation from multiple replications of treatments. Different small letters indicate significant different means (*p* < 0.05) within different OD trials.

In the present study, in order to evaluate the effectiveness of a reconstituted OD solution in WL, SG and a_w_ of the figs, four consecutive trials of OD at optimal conditions (45 °C, 90 min) were conducted using a 3-times reconstituted OD solution. At the end of the first trial, WL, SG and a_w_ values of figs were estimated and found to be equal to 2.170 g H_2_O/g i.d.m., 0.190 g s./g i.d.m. and 0.9214, respectively. The corresponding values at the end of the 4th trial were 2.145 g H_2_O/g i.d.m., 0.174 g s./g i.d.m. and 0.9253, respectively. No significant differences were detected on the effectiveness of the trials (*p* > 0.05) (Figure 3).

The main osmotic agent (80%) of OD solution was food-grade glycerol. Its low molecular weight resulted in a significant reduction of water activity of the OD solution, approximately equal to 0.48. After 90 min of OD treatment at 45 °C, the water activity of OD solution slightly increased and reached the value of ~0.54. The repetitive reconstitution of OD solution resulted in a decrease of its water activity in initial value (~0.48). The low water activity seems to have inhibited adequately the microbial growth. However, considering that during OD many organic components, released to OD solution, are able to act as substrates for microbial growth, microbiological analysis of the OD solution was conducted after the 4th trial, in order to evaluate the potential of re-using and recycling the OD solution for many cycles. Concerning the microbiological analysis performed, it is well-known that some osmo-tolerant or osmo-philic microorganisms are resistant to high sugar concentrations, causing major issues in industrial recycling of OD solution. Loads of the TVC and yeasts/molds in the OD solution were below the detection limit (<2 logCFU/g),which could be attributed to the low water activity of OD solution that ensures its microbiological stability and to the short processing time (90 min for each batch) that is not sufficient for observable microbial growth.

Thermal treatment of OD solution is recommended after 5 cycles of OD use, ensuring microbiological stability of OD process and not cross-contamination between different processed batches, thus enabling the reuse of OD solution as many times as possible. The reconstitution of OD solution is a need concerning industrial scale-up of OD-technique, since it will address the environmental concerns of discarding the OD solutions, while simultaneously will result in final products cost reduction.

Garcıa-Martınez, Martınez-Monzó, Camacho, and Martınez-Navarrete [42], studied the effect of the OD on kiwi fruit and reported that the reuse of the OD solution was effective for at least 10-times without issues related to fruit dehydration level or microbiological contamination. Valdez-Fragoso, Mujica-Paz, Giroux, and Welti-Chanes [43] after reusing the OD solution for up to six times, estimated similar effectiveness to those of the first trial for OD-treated apples.

### 3.2. Air-Drying Processing of Osmotic Dehydrated and Untreated Figs

**Figure 4:**
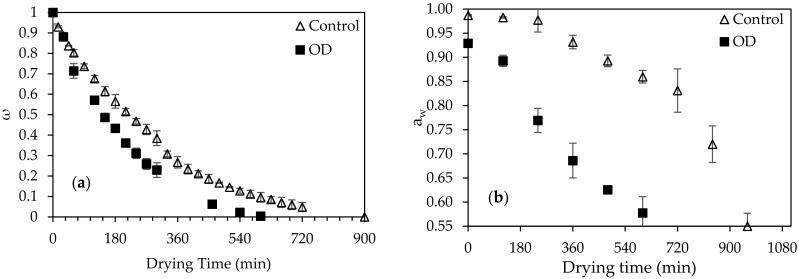
Effect of air-drying at 70 °C on the (**a**) non-dimensional moisture content ω and (**b**) water activity a_w_, of the OD pretreated (45 °C, 90 min, 1/4 ratio of sample per OD solution (*w_s_*/*w_OD_*)) (■) and untreated (Control) (△) figs. Error bars represent standard deviation from multiple replications of treatments.

The effect of air-drying temperatures equal to 50, 60 and 70 °C on the drying kinetics of the OD-pretreated (at the optimal conditions) and untreated fig halves, was studied (Figure 4a,b).

Increase of the air-drying temperature significantly enhanced the water removal from the figs (*p* < 0.05), as expected [44].

Based on the Fick’s 2nd law, the apparent diffusion coefficients (*D_eff_*) for the drying temperatures studied, were calculated considering as 0.55 the a_w_-value of the final dried figs (Table 2).

At the drying temperatures studied, OD-pretreated figs showed significantly higher *D_eff_* values compared to the control counterparts (*p* < 0.05). Da Costa Ribeiro et al. [11], observed significantly higher rates in water removal of OD-pretreated pears compared to those of untreated samples. The *D_eff_* values of the OD-pretreated and untreated figs were estimated in the ranges of 0.77–1.21 × 10^−10^ and 0.55–0.95 × 10^−10^ m^2^ s^−1^, respectively. Şahin and Öztürk [24], studied the air-drying process of OD-pretreated and untreated figs and reported *D_eff_* values ranging from 3.57 × 10^−10^ to 10.25 × 10^−10^ and from 2.75 × 10^−10^ to 5.69 × 10^−10^ m^2^ s^−1^, respectively. In literature, there are also other mathematical models that have been used to describe OD and drying kinetics of figs, with a satisfactory fitting on the experimental data, such as *Peleg* and *Azuara* model [9] and a proposed thin layer drying model [2]. The effect of temperature on the *D_eff_* values was expressed through the *E_a_* (Equation (3)). OD-pretreated samples presented lower *E_a_* value compared to the respective untreated, equal to 20.80 ± 2.56 and 25.34 ± 1.20 kJ/mol, respectively, expressing less temperature dependence on the OD drying rate of the figs, compared to the use of stand-alone air-drying.

Aiming at assuring microbial safety and reducing the non-enzymatic hydrolysis and Maillard reaction, the final a_w_ was selected to be equal to 0.55. OD pretreatment led to a significant reduction of the initial a_w_ (from 0.9870 to 0.9292), resulting in significant decrease of air-drying time (*p* < 0.05) in order to achieve the final desired a_w_-value (0.55) for “shelf-stable” products. The drying time needed for the OD-treated and untreated figs was 11.2 and 16.1 h, respectively, at 70 °C, showing a significant reduction in the air-drying time by approximately 30.4%. For milder drying temperatures (60 and 50 °C), the air-drying time of the OD-pretreated figs was reduced approximately by half compared to untreated samples (Table 3).

Dermesonlouoglou et al. [12] observed that the air-drying times of OD-pretreated tomato and cucumber were significantly reduced up to 28 and 47%, respectively, when compared to control. Moreover, Dermesonlouoglou et al. [13] reported that the OD-pretreatment of goji berries followed by air-drying, led to a decrease of drying time by 120 min.

### 3.3. Energy Savings and Yield Increase of the Combined Use of Osmotic Dehydration and Air-Drying on Figs

OD-pretreatment led to a significant reduction of the initial a_w_, resulting in a significantly decreased air-drying processing time (*p* < 0.05) of approximately 50%, for achieving the final a_w_-value equal to 0.55 for “shelf-stable” products. The decrease in the air-drying time, could lead to a final product of increased quality, as well as to increased energy savings. Based on Equation (9), the energy consumption of the untreated and OD-pretreated samples for air-drying at 60 °C was 480 and 331 MJ kg^−1^, respectively. When OD pre-treatment was applied, the energy consumption for air-drying at 50, 60, and 70 °C was by 42.5, 31.1, and 18.0% less than the respective energy required for untreated samples. The most pronounced energy savings was observed for drying at 50 °C, estimated as 177 MJ kg^−1^. Alibas [45], estimated a 3-fold decrease in the energy consumption for pumpkin slices using a combined technique of microwave and air-drying, compared to the conventional air-drying.

Based on data received for product weight measurements before and after processing, the yield of the OD-pretreated final product was higher, since for the production of 1 kg of final dried figs, 4 kg of control samples should be dried, in contrary to 2 kg of OD-treated samples (100% yield increase). These results sustain and emphasize the benefits of OD as a pretreatment for drying of fig halves, making it an attractive and feasible approach for implementation in an industrial drying process line.

Based on the data received during air-drying and taking into account the no-significant differences between the estimated required time for air-drying at 60 and 70 °C (*p* > 0.05) (Table 3) and the reduced energy consumption at lower temperatures, the optimal condition selected was 60 °C.

### 3.4. Cost-Effectiveness Analysis of OD-Assisted Air Drying of Fig Halves

A cost analysis approach was performed, estimating the total cost of OD process at the selected optimal conditions (45 °C, 90 min, 1/4 (*w_s_*/*w_OD_*) (and formulation of OD solution: 80% glycerol, 1% salt, 1% vinegar, 0.5% ascorbic acid and 17.5% water). The costs for the OD ingredients, the energy consumption during OD and unit operation costs were provided by collaborating suppliers and industries, respectively. It was estimated that the OD solution cost would be approximately to 0.63 € kg^−1^ fresh fig, assuming that the reconstitution of OD solution would be at least 4 times (as also suggested in our study). The energy consumption during OD (90 min, 45 °C) was estimated as 0.021€ kg^−1^ fresh fig when industrial scale operation units were used. The total cost of the OD process was estimated as approximately 0.65 € kg^−1^ fresh fig.

The cost for the conventional and OD-assisted air drying at 60 °C was calculated based on the energy consumption (kWh) of industrial equipment (considering a cost of 0.06 € kWh^−1^). According to the obtained results (Section 3.2), the drying time was approximately 16 and 11 h for the conventional and OD-assisted air-drying process, that correspond to 1.18 and 0.67 € kg^−1^ fresh fig, respectively. Considering also that in OD-assisted drying the yield of the product was increased (for the production of 1 kg of dried figs, 2 kg of OD pre-treated figs are needed or 4 kg of conventionally air-dried figs) the total cost of conventional air drying was estimated as more than 3-fold the cost per kg for OD pre-treated figs.

### 3.5. Quality Evaluation of Osmotic Pretreated and Untreated Air-Dried Figs

Quality characteristics of the OD-pretreated (45 °C, 90 min, 1/4 (*w_s_*/*w_OD_*)) air-dried figs, processed at the optimal drying conditions (60 °C), were evaluated, and compared to those of conventionally air-dried figs (Table 4).

OD-pretreatment led to the production of dried figs of improved quality characteristics such as brighter color and softer texture due to the significant reduction of the required air-drying time, as well as to structural changes that occur in the food surface during OD. The firmness of the OD-pretreated dried figs was decreased by up to 40% (18.84 N) compared to the non-pretreated ones (31.84 N). According to Yadav and Singh [46], OD-pretreatment helps the fruit structure to be unaffected during the subsequent drying, mainly due to structural changes caused in the waxy layer of the fruit surface during OD. Mandala et al. [14] have reported that OD-pretreatment of apple slices using a glucose and sucrose (30%) solution, resulted in softer surface during drying. Shamaei, Emam-Djomeh, and Moini [47] also showed that OD and air-dried cranberries had a softer texture compared to untreated samples, due to the use of lower drying temperature and reduced drying time.

Regarding the luminosity (L*-value) of the flesh, for the OD-pretreated figs a slight increase was observed increased compared to the non-pretreated ones, however this was not perceived during the sensory evaluation. Based on the estimated L*, a* and b*-parameters, OD-pretreatment did not cause any significant color changes to the fig halves (*p* > 0.05) (Table 4).

Mandala et al. [14] reported that OD pretreatment (45% sugar solution) led to color retention of apple slices during drying. Da Costa Ribeiro et al. [11] proved that the combination of OD and conventional drying resulted in a 17% higher color acceptability than the one obtained by the conventionally dried pears. Dermesonlouoglou et al. [12] observed that OD-treated, air-dried goji berry retained their color characteristics when drying time was decreased, compared to untreated samples.

Sensory properties, such as color, odor, flavor, texture and taste of both dried figs, were also examined. The odor and flavor of the untreated dried fig halves were scored lower than OD-pretreated samples, due to the loss of volatile organic compounds during the time-consuming air-drying [48]. El-Gendy [21] reported that the OD-pretreatment of figs with 70% syrup, resulted in increased scores of sensory characteristics, compared to the untreated counterparts.

The nutritional and bioactive compounds of both OD-pretreated and control dried fig halves, were also measured (Table 5), in order to assess the impact of the OD process on their nutritional profile. OD-pretreated samples had significantly increased content of intracellular compounds, compared to the control samples (*p* < 0.05).

The concentration in total phenolics and flavonoids of the untreated air-dried figs was estimated as 22.31 mg CAE/100 g d.w. and 4.47 mg catechin/100 g d.w, respectively. The use of OD increased significantly (*p* < 0.05) the concentration of both total phenolics and flavonoids bioactive compounds by 18% (26.34 mg CAE/100 g d.w.) and 15.4% (5.16 mg catechin/100 g d.w.), respectively. The higher retention of the intracellular bioactive compounds for the OD-pretreated figs could be attributed to the reduced oxidation reactions due to the shorter duration of air-drying (9.2 h less compared to control samples) or/and due to the use of OD solution that has a protective effect on exposure of fig in oxygen. OD-pretreated dried figs presented a significantly (*p* < 0.05) higher antioxidant activity (14.18 mg Trolox/100 g d.w.) compared to the control ones (13.14 mg Trolox/100 g d.w.), due to their increased bioactive compounds content.

No significant differences were observed in total fibers content for the OD-pretreated and untreated dried fig halves, estimated as 10.5 and 8.5 g/100 g d.w., respectively. Similarly, to this result, El-Gendy [21] reported slight increase of fibers in OD-treated dried fig halves by ~4.5%, compared to control.

No significant differences were observed for the proteins and the main sugars (glucose and fructose) of both conventionally air-dried and OD pretreated-air-dried figs (*p* > 0.05). OD-pretreated dried figs contained an amount of 3.0% glycerol, mainly attributed to the solid uptake of the fig halves during OD. At the optimal OD conditions, it was estimated that the SG was equal to 0.09 g/g d.w. after 90 min of OD. During OD, the diffusion phenomenon of solids took place with two countercurrent flows: a major solute flow from the OD solution to the fig halves, enriching its nutritional value, and a minor simultaneous flow of solute from the fig halves to OD solution, decreasing the concentration of some soluble solids that leached into the OD solution. The polyvalent organic acids measured were found to be significantly decreased for the OD-pretreated dried figs (*p* < 0.05) compared to the control dried samples, due to solute transfer from the fig into the OD solution. Phisut, Rattanawedee, and Aekkasak [49] concluded that during OD treatment, natural solutes such as acids, vitamins, and small molecules were extracted from the fruit into the OD solution. The content of the OD-pretreated dried samples in ascorbic acid was estimated as 15.61 mg/100 g d.w., ~50% decreased compared to the untreated samples. The main mechanisms of the loss in vitamin C, appears to be due to its water solubility, mass transferability (leaching out of the vegetative cell), and heat sensitivity.

### 3.6. Shelf-Life Determination

**Figure 5:**
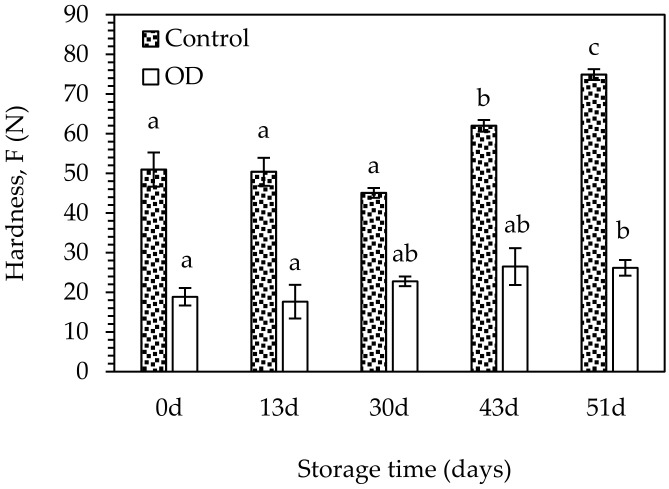
Hardness (F) of the control and OD-pretreated air-dried fig halves during storage at 45 °C. Error bars represent standard deviation from multiple replications of treatments. Different small letters indicate significantly different means (*p* < 0.05) between storage time (days).

In order to estimate the shelf-life of both pre-treated and untreated air-dried figs, an accelerated experiment was conducted, including the monitoring of quality parameters and sensory evaluation of samples during storage at 25, 35, and 45 °C for ~2 months. The hardness of all fig halves increased during storage, deviating significantly from their initial value for storage times longer than 30 and 51 days at 45 °C, for OD-pretreated and untreated samples, respectively (Figure 5).

OD-pretreatment led to a better retention of the hardness of the already improved fig texture (softer), during storage, leading to a value of approximately 26.17 N after 51-days of storage at 45 °C, instead of 74.89 N for the control. Based on measurements of the hardness during the sensory evaluation, the OD-pretreated figs received higher scores compared to the untreated ones throughout the whole storage period.

**Figure 6:**
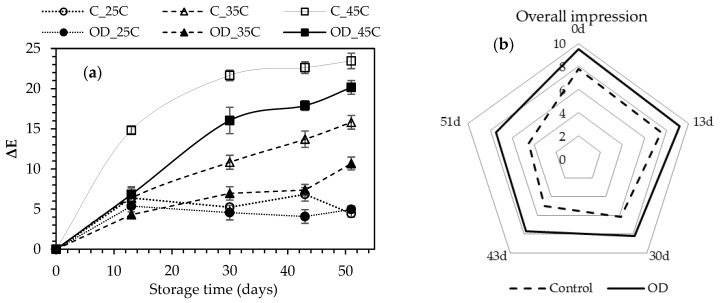
(**a**) Color change (Δ*Ε*) and (**b**) Overall impression for the control and OD-pretreated air-dried fig halves during storage at 25 °C, 35 °C and 45 °C. Error bars represent standard deviation from multiple replications of treatments.

Despite the increase of fig hardness, their color was considered by the organoleptic panel to be the main parameter for the sensory rejection of the product. In all cases, increase of storage temperature led to increased color change (Δ*Ε*), mainly attributed to the non-enzymatic browning that took place (*Maillard* reaction) (Figure 6).

**Figure 7:**
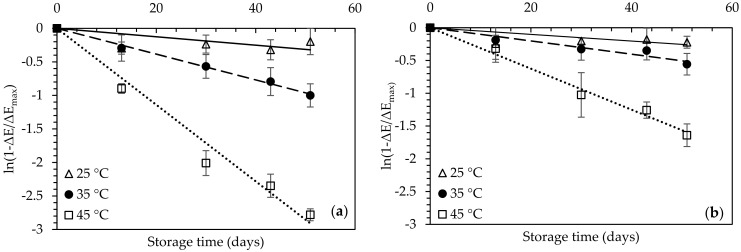
Kinetic modelling of the effect of the storage time and temperature (25 °C (△), 35 °C (⚫) and 45 °C (☐)) on the color change of the (**a**) Control and (**b**) OD-pretreated air-dried fig halves. Error bars represent standard deviation from multiple replications of treatments.

Increase in storage temperature led to increased rate constants of the color change for the untreated dried figs (Figure 7) showing significantly higher values compared to the OD-pretreated ones (*p* < 0.05) (Table 6).

The effect of storage temperature on the rate constants of the fig color change was expressed through the activation energy (*E_a_*), calculated for both OD-pretreated and untreated dried figs as 93.6 and 79.9 kJ mol^−1^, respectively (Equation (8)).

The acceptability of the dried figs was expressed through an average sensory score of 5 which was found to be well-correlated to a color change value (Δ*Ε*) of 20. The shelf-life of the control and the OD-pretreated figs for storage at 25 °C, was estimated through extrapolation of the linear regression of Equation (11) as 9.9 and 13.7 months, respectively.

## 4. Conclusions

Applying more intense OD conditions led to accelerated solid and water transfer. The optimal OD processing conditions selected were 45 °C, a processing time of 90 min, and 1/4 ratio (*w_s_*/*w_OD_*) for an adequate mass transfer of WL: 2.17 g w/g i.d.m., SG: 0.19 g s/g i.d.m. and an a_w_ decrease from ~0.986 to ~0.929. OD-pretreatment led to a significant reduction of the required air-drying time at 60 °C, resulting in high energy savings, estimated as 150 MJ kg^−1^, while at the same time the quality, nutritional value and organoleptic properties were significantly improved. The application of OD-pretreatment prior to air-drying led to better color retention and tissue softening, decreasing the firmness by 70% compared to the untreated sample. During the subsequent shelf-life study, the OD-pretreated figs presented a better retention of their color and texture characteristics, yielding the highest scores during the sensory evaluation, compared to figs of conventional air-drying. The shelf-life for the control and the OD-pretreated dried figs was estimated as 9.9 and 13.7 months at 25 °C, respectively. The results confirm the significant benefits of OD process as a pretreatment of air-drying, making it an alternative efficient approach for industrial implementation in vegetable and fruit drying process lines with the possibility of reuse of the osmotic solution.

## Figures and Tables

**Table 1:** Diffusion coefficients for water loss (*D_ew_*) and solid gain (*D_es_*) during OD processing of figs for processing temperatures 25–45 °C and ratio of sample per OD solution 1/4 and 1/6 (*w_s_*/*w_OD_*) for 80% glycerol concentration.

T (°C)	*D_ew_* (m^2^ s^−1^)		*D_es_* (m^2^ s^−1^)	
	Ratio 1/4	Ratio 1/6	Ratio 1/4	Ratio 1/6
25	0.94 × 10^−10^ ± 1.05 × 10^−11 Aa^	0.95 × 10^−10^ ± 1.73 × 10^−11 Aa^	0.56 × 10^−10^ ± 1.40 × 10^−11 Aa^	0.43 × 10^−10^ ± 1.65 × 10^−8 Aa^
35	1.66 × 10^−10^ ± 1.35 × 10^−11 Ba^	1.63 × 10^−10^ ± 3.53 × 10^−11 Ba^	1.19 × 10^−10^ ± 2.32 × 10^−11 Ba^	1.12 × 10^−10^ ± 1.14 × 10^−11 Ba^
45	2.10 × 10^−10^ ± 1.67 × 10^−11 Ca^	2.11 × 10^−10^ ± 3.28 × 10^−11 Ca^	1.53 × 10^−10^ ± 2.94 × 10^−11 Ca^	1.48 × 10^−7^ ± 2.59 × 10^−11 Ca^

± represents the standard error of nonlinear regression analysis. Different superscripts indicate significant different means (*p* < 0.05) within different temperatures (letters in capital) or between the different ratios (*w_s_*/*w_OD_*) (small letters).

**Table 2:** Diffusion coefficients (*D_eff_*) at drying processing temperatures 50, 60, 70 °C of air-dried OD pretreated (45 °C, 90 min, 1/4 ratio of sample per OD solution (*w_s_*/*w_OD_*)) and untreated (control) figs.

T (°C)	*D_eff_* (m^2^ s^−1^)	
	Control	OD
70	0.95 × 10^−10^ ± 1.05 × 10^−11 a^	1.21 × 10^−10^ ± 1.73 × 10^−11 a^
60	0.88 × 10^−10^ ± 1.35 × 10^−11 a^	0.97 × 10^−10^ ± 0.53 × 10^−11 a^
50	0.55 × 10^−10^ ± 0.67 × 10^−11 b^	0.77 × 10^−10^ ± 0.28 × 10^−11 b^

± represents the standard error of nonlinear regression analysis. Different superscript small letters indicate significantly different means (*p* < 0.05) within different drying temperatures (data in a row).

**Table 3:** Total air-drying time for control and OD-pretreated fig halves at 50, 60, and 70 °C drying temperature.

	Drying Temperature (°C)		
T_DR_ (h)	50 °C	60 °C	70 °C
Control	36.5 ± 1.2 ^aA^	21.2 ± 0.8 ^bA^	16.1 ± 0.8 ^cC^
OD	19.1 ± 2.4 ^aB^	12.0 ± 1.5 ^bB^	11.2 ± 0.9 ^bB^

± represents the standard error of nonlinear regression analysis. Different superscript capital letters indicate significantly different means (*p* < 0.05) within a column (differences between OD and untreated samples) and different superscript small letters indicate significantly different means (*p* < 0.05) within a row (differences between drying temperatures).

**Table 4:** Firmness (N) and color (L*, a*, b*) of OD-pretreated (45 °C, 90 min, 1/4 ratio of sample per OD solution (*w_s_*/*w_OD_*)) and untreated dried fig halves.

Quality Parameters	OD-Pretreated Figs	Untreated Figs
Firmness (N)	18.87 ± 2.84 ^a^	31.84 ± 4.14 ^b^
Color (flesh)		
L*	29.61 ± 1.58 ^a^	33.03 ± 1.98 ^b^
a*	14.24 ± 2.33 ^a^	14.09 ± 3.25 ^a^
b*	19.93 ± 2.51 ^a^	18.82 ± 3.17 ^a^
Color (skin)		
L*	25.36 ± 3.28 ^a^	24.47 ± 2.84 ^a^
a*	3.99 ± 0.89 ^a^	4.17 ± 0.91 ^a^
b*	6.44 ± 2.86 ^a^	6.83 ± 3.15 ^a^

± represents standard deviation from multiple replications of treatments. Different superscript small letters indicate significantly different means (*p* < 0.05) between OD and untreated dried samples.

**Table 5:** Nutritional and bioactive compounds of OD pretreated (45 °C, 90 min, 1/4 ratio of sample per OD solution (*w_s_*/*w_OD_*)) and untreated dried figs.

Nutritional Parameter	OD-Pretreated Figs	Untreated Figs
Total phenolic content (mg CAE/100 g d.w.)	26.34 ± 0.25 ^b^	22.31 ± 0.26 ^a^
Total flavonoids (mg catechin/100 g d.w.)	5.16 ± 0.33 ^b^	4.47 ± 0.11 ^a^
Antioxidant capacity (mg Trolox/100 g d.w.)	14.18 ± 0.16 ^b^	13.14 ± 0.05 ^a^
Total fibers (g/100 g d.w.)	10.50 ± 0.85 ^a^	8.52 ± 1.22 ^a^
Proteins (g/100 g d.w.)	9.33 ± 0.82 ^a^	11.01 ± 0.95 ^a^
Glucose (mg/100 g d.w.)	0.73 ± 0.05 ^b^	0.64 ± 0.08 ^a^
Fructose (mg/100 g d.w.)	1.43 ± 0.02 ^b^	1.25 ± 0.06 ^a^
Glycerol (mg/100 g d.w.)	3.00 ± 0.56 ^a^	-
Ascorbic acid (mg/100 g d.w.)	15.61 ± 3.61 ^a^	33.94 ± 2.21 ^b^
Citric acid (mg/g d.w.)	9.20 ± 1.12 ^a^	15.8 ± 0.25 ^b^
Tartaric acid (mg/100 g d.w.)	1.65 ± 0.28 ^a^	3.03 ± 0.91 ^b^
Malic acid (mg/100 g d.w.)	2.75 ± 0.17 ^a^	8.15 ± 1.52 ^b^
Lactic acid (mg/100 g d.w.)	0.72 ± 0.12 ^a^	1.12 ± 0.22 ^b^

± represents standard deviation from multiple replications of treatments. Different superscripts indicate significantly different means (*p* < 0.05) between OD and untreated dried samples.

**Table 6:** Kinetic characteristics of the color change, rate constants k (d^−1^) and activation energies E_a_ (kJ mol^−1^) of the control and OD-pretreated dried figs during storage at 25–45 °C and shelf-life determination (d) at 25 °C.

Storage Temperature (°C)	K (d^−1^)	
	Control	OD
25	0.0053 ± 0.0005 ^aA^	0.0041 ± 0.0004 ^aB^
35	0.0192 ± 0.0012 ^bA^	0.0101 ± 0.0025 ^bB^
45	0.0570 ± 0.0095 ^cA^	0.0313 ± 0.0087 ^bB^
E_a_ (kJ mol^−1^)	93.6 ± 3.6 ^A^	79.9 ± 5.6 ^B^
Shelf-life determination at 25 °C (d)	297 ± 15 ^A^	411 ± 22 ^B^

± represents the standard error of nonlinear regression analysis of Equations (10) and (11). Different superscript capital letters indicate significantly different means (*p* < 0.05) within a row (differences between OD and untreated samples) and different superscript small letters indicate significantly different means (*p* < 0.05) within a column (differences between storage temperatures).

## Data Availability

The datasets generated during the current study are available from the corresponding author on reasonable request.
